# Supplementary material for: Splice-Junction-Based Mapping of Alternative Isoforms in the Human Proteome
Source: Cell Rep. Author manuscript; Available in PMC 2020 Jan 15. (PMC6961840; doi:10.1016/j.celrep.2019.11.026)
Supplement: 2 [file NIHMS1546469-supplement-2.zip › DF1/PXD006675/RightVentricle/RightVentricle_42_BBS1_EGQSAPLLSAHVNMPGSEGLAAPNRPLNPE.pdf]

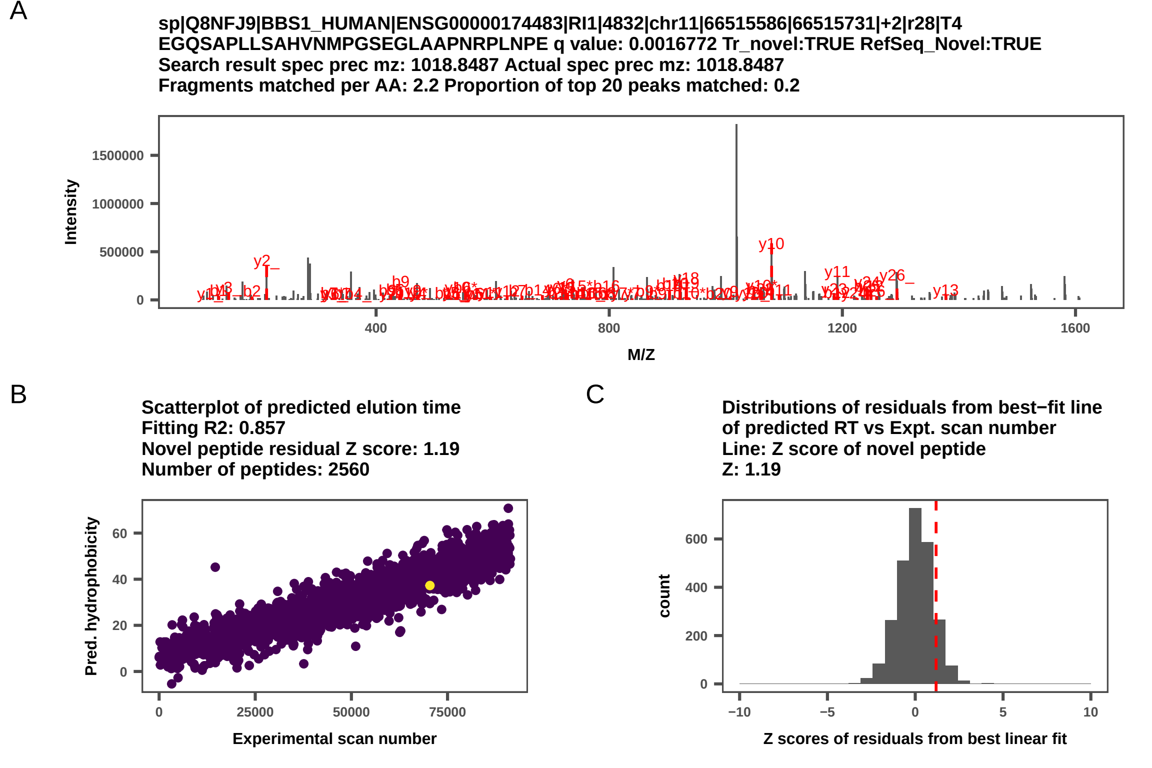

A
sp|Q8NFJ9|BBS1_HUMAN|ENSG00000174483|RI1|4832|chr11|66515586|66515731|+2|r28|T4
EGQSAPLLSAHVNMPGSEGLAAPNRPLNPE q value: 0.0016772 Tr_novel:TRUE RefSeq_Novel:TRUE
Search result spec prec mz: 1018.8487 Actual spec prec mz: 1018.8487
Fragments matched per AA: 2.2 Proportion of top 20 peaks matched: 0.2
Intensity
1500000
1000000
500000
0
400
800
1200
1600
M/Z
y2_
y10
y11
y26_
y13
y18
b9
B
Scatterplot of predicted elution time
Fitting R2: 0.857
Novel peptide residual Z score: 1.19
Number of peptides: 2560
Pred. hydrophobicity
60
40
20
0
0
25000
50000
75000
Experimental scan number
C
Distributions of residuals from best−fit line
of predicted RT vs Expt. scan number
Line: Z score of novel peptide
Z: 1.19
count
600
400
200
0
−10
−5
0
5
10
Z scores of residuals from best linear fit
